# Barriers and Facilitators to Data Use for Decision Making: The Experience of the African Health Initiative Partnerships in Ethiopia, Ghana, and Mozambique

**DOI:** 10.9745/GHSP-D-21-00666

**Published:** 2022-09-15

**Authors:** African Health Initiative Partnership Collaborative for Data Use for Decision Making

**Affiliations:** a Members listed at the end of the article.

## Abstract

**Background:**

Three African Health Initiative (AHI) partnership projects in Ethiopia, Ghana, and Mozambique implemented strategies to improve the quality and evaluation of routinely collected data at the primary health care level and stimulate its use in evidence-based decision making. We compare how these programs designed and carried out data for decision-making (DDM) strategies, elaborate on barriers and facilitators to implementation success, and offer recommendations for future DDM programming.

**Methods:**

Researchers from each project collaboratively wrote a cross-country protocol based on these objectives. By adapting the Consolidated Framework for Implementation Research (CFIR) through a qualitative theme reduction process, they harmonized lines of inquiry on the design of the respective DDM strategies and the barriers and facilitators of effective implementation. We conducted in-depth interviews and focus group discussions with stakeholders from the primary health care systems in each country, and we carried out multistage, thematic analyses using a deductive lens.

**Results:**

Effective implementation of DDM depended on whether implementers felt that DDM was adaptable to context, feasible to trial, and easy to introduce and maintain. The prevailing policy and political environment in the wider health system, learning climate and absorptive capacity for evidence-based change in DDM settings, engagement of external change agents and internal change leaders, and promotion of opportunities and means for team-based reflection and evaluations of what works influenced the success or failure of DDM strategies.

**Conclusion:**

Opportunities for team-based capacity building and individual mentorship led to effective DDM programming. External policies and associated incentives bolstered this but occasionally led to unintended consequences. Leadership engagement and availability of resources to act on recommendations; respond to capacity-building needs; and facilitate collaborations between peers, within hierarchies, and across the local health system proved crucial to DDM, as was encouraging adaptation and opportunities for iterative on-the-job learning.

## Abstract (precis)

Data for decision making on clinical care and health service management is crucial, yet implementers lack knowledge on the determinants of effective implementation. Findings from this study conducted in the context of primary health care systems in Ethiopia, Ghana, and Mozambique fill this knowledge gap.

## Teaser

**Key Findings:**

- Creating regular meeting time and space for team-level capacity building through mentorship, joint analysis of data, and results sharing bolsters sustainable engagement in data use for decision making at the local level.
- External policy and associated incentives led to broad enthusiasm and facility-level operational challenges.
- Leadership engagement and available resources, at the facility and subnational levels, drove implementation readiness across projects. Performance lagged in settings where leadership felt underprepared to support state initiatives to expand health management and information systems. Where training and subsequent mentorship were farther reaching across health-system levels, impact on service delivery was greater.

**Key Implications:**

- To effectively implement sustained improvement interventions for data for decision making, health facility managers should create or build into existing team meeting spaces the time for capacity building through joint data sharing, analysis, and improvement programming.
- National and subnational managers should prioritize engagement and capacity across health systems levels through training opportunities and mentorship. Likewise, policies to improve the overall skills of the workforce should be considered at both the individual and operational levels.

Resumo em português no final do artigo.

## INTRODUCTION

Data generated from health management information systems (HMIS) in low- and middle-income countries (LMICs) are not used consistently across all health-system levels.^1^^,^^2^ In multiple settings, it has been shown that while improving HMIS data quality and use in LMIC settings is possible, their subsequent use to inform decision making is often stymied.^3^^,^^4^ Innovative and appropriately contextualized strategies to improve data use across health systems—in community, clinical, and management settings—are critical to bridging the gap between what we know and what we do (the “know-do gap”) in health service delivery, especially in under-resourced settings.^5^ Strategies that encourage data use at the community level, including outreach and doorstep strategies for child survival and HIV-related and family planning interventions, promote accountability of local health systems to community priorities.^6^^,^^7^ At the facility level, data-use interventions that emphasize “audit and feedback” using routine data systems are known to improve clinical care and professional practice.^8^^–^^11^ Likewise, successful management strategies to promote data utilization at the district level in LMICs focus on identifying and tailoring priorities to local burden of disease, budget preparations, and improvements in population coverage planning.^12^^–^^16^ Thus, addressing the determinants of effective utilization of routine health data is critical for promoting broader strengthening of health systems in LMICs.

Addressing the determinants of effective utilization of routine health data is critical for promoting broader strengthening of health systems in LMICs.

Studies have noted that HMIS designs often lack standardized and adaptive processes for data use across multiple geographic and clinical settings.^17^^,^^18^ Other research has elaborated on adverse contextual factors that undermine the quality and completeness of clinical and service delivery data in LMICs and also disincentivize an overall culture of data use.^9^^,^^19^^–^^21^ Organizations’ potential to benefit from data utilization is often constrained by local perceptions that data gatherers and users are 2 distinct groups.^22^ This bifurcation in perceived roles and responsibilities contributes to the lopsided development of large data collection enterprises alongside underdeveloped data analysis and use by health policy makers, managers, and other key stakeholders.^4^^,^^20^^,^^23^ Fostering the use of data during decision making, for both clinical and administrative services, is essential for prioritization, planning, managing health care workers, and introducing new services, as well as improving existing ones, to better meet the needs of local populations and the teams who serve them.^24^ Thus, identifying ways to translate data-based findings into practice is a foremost priority for researchers, policy makers, and practitioners.^25^

### Linking Data for Decision-Making Strategies to Evidence-Based Interventions in 3 Countries

The African Health Initiative (AHI) of the Doris Duke Charitable Foundation has supported partnership programs in Ethiopia, Ghana, and Mozambique to promote and sustain HMIS data utilization for decision making. This work aims to help achieve broader coverage, higher quality, and longer sustainment of distinct, evidence-based primary health care-related interventions—thereby achieving large-scale improvements in population health, largely focusing on maternal neonatal child health (MNCH) care services. Each country project identified a broad, evidence-based intervention (EBI) currently supported by their respective Ministry of Health (MOH). The projects then linked their data for decision-making (DDM) strategies to this EBI (Supplement Table). While DDM interventions across the 3 country projects were not designed to have commonalities, some occurred organically. To bolster both ownership and sustainability of the work, each project also invested in capacity building of local researchers who partner with district, regional, and national-level health systems.

In Ethiopia, the MOH launched the Connected Woreda strategy in 2016 to support the government’s broader Information Revolution Initiative.^26^^,^^27^ The goal of this strategy is to transform and enhance the culture of data use through fostering evidence-based decision making in 3 subcities of Addis Ababa (Yeka, Akaki-Kaliti, and Ledeta). Much of this strategy depends on creating a culture of data use by improving methods and practices in data analysis and use; enhancing access to and visibility of routine data; establishing an interoperable architecture to foster integration, standardization, and harmonization across data sources and health information systems; and deploying appropriate technologies to strengthen all aspects of data use. By improving access to and use of quality health information for decision making across all levels, Connected Woreda supports the delivery of quality and equitable health services.

In Ghana, the targeted EBI was the national Community-based Health Planning and Services (CHPS) program. Through CHPS, community health officers are deployed in communities and work with community health volunteers to deliver primary health care services. Since 2015, AHI partners in Ghana have implemented the CHPS+ Project, which comprises a suite of interventions to scale up the national program, CHPS, from 4 system learning districts to 2 additional regions. With respect to DDM, the CHPS+ Project supported local authorities in the roll-out of the e-Tracker, a digital dashboard for collecting point-of-care service data to help empower primary health care workers in service activity planning by providing service utilization trends in “real time.”

In Mozambique, the target EBI was the MOH’s MNCH guidelines. With Doris Duke Charitable Foundation support, AHI partners employed an iterative, 3-step process of data-driven, primary health care systems strengthening in 12 districts in the central region of Mozambique to scale up the use of the national MNCH guidelines by primary health care teams. The 3-step process consisted of (1) health systems and facility readiness assessments, (2) blended district- and facility-level audit and feedback, and (3) targeted supportive supervision coupled with financial support.

Our goal was to learn from the experiences of AHI partners in Ethiopia, Ghana, and Mozambique by examining their DDM implementation strategies and the factors that shaped their effectiveness. To this end, we employed theoretical frameworks developed to help document implementation strategies and the determinants of implementation effectiveness across these heterogeneous settings.^28^^–^^32^ Specifically, we (1) compare how AHI-funded programs designed and carried out DDM strategies in local health systems, (2) rigorously assess the barriers and facilitators to the successful implementation of DDM strategies, and (3) offer recommendations and insights for future DDM programming. Note that while successful implementation of DDM strategies was defined by each specific project, all definitions of success shared the overarching goal of promoting pri-mary health care worker data analysis, which then linked to their self-reported use of the data for decision making.

Our goal was to learn from the experiences of AHI partners in Ethiopia, Ghana, and Mozambique by examining their DDM implementation strategies and the factors that shaped their effectiveness.

## METHODS

Our “AHI DDM working group” included representatives from each project, a working group lead, and a working group coordinator; it commenced efforts by a series of exchanges between May 2019 and December 2019. Our correspondence led to a concept paper and 3 project-specific study protocols. During this phase, we defined DDM intervention strategies in terms of the study teams’ targeted efforts to support the implementation of identified EBIs (e.g., Ethiopia CWS, Ghana CHPS+, and Mozambique MNCH MOH guidelines). Across the 3 country teams, we jointly defined effective data use resulting from DDM intervention strategies as either primary health care or community level health care teams producing, analyzing, and using routine data to generate performance information and guide health system planning, per self-report of respondents. We used Proctor’s framework for specifying implementation strategies to summarize and organize the core components and objectives of each project’s DDM strategies.^30^

We used 2 implementation science frameworks to develop data collection tools and guide data collection. Specifically, insights “from classification to causality” in implementation science were used to identify the main intervention strategies, their characteristics, and the causal pathways through which those strategies and characteristics influence data usage.^33^ Those characteristics and influences were categorized under constructs from the Consolidated Framework for Implementation Research (CFIR).^34^^,^^35^ We used both a case-oriented analysis, which treated each district as a unique case, and an extended case-study approach to assess connections, commonalities, and differences across the districts.^36^^,^^37^ Table 1 provides detail on the participant sampling carried out in each country.

**TABLE 1:** In-Depth Interview and Focus Group Discussion Recruitment Sample, by African Health Initiative Project

	**In-Depth Interview**		**Focus Group Discussion**	
	No.	Participants	No.	Participants
Ethiopia	40	Regional and woreda-level health management team members; health center staff (head, deputy head, health information technicians, and maternal/child health care workers)	43	Regional and woreda-level health management team members; health center staff (head, deputy head, health information technicians, and maternal/child health care workers), university staff
Ghana	34	District health management team and community health officers, Ghana Health Service	52	Community members, community health volunteers, subdistrict health team
Mozambique	7	Chief district medical officers, district maternal child health supervisors	39	Maternal/child health nurses

Between November 2019 and June 2020, AHI programs collected primary data that included qualitative in-depth interviews (IDIs) and focus group discussions (FGDs) with key informants from various levels of the health systems in Ethiopia, Ghana, and Mozambique. In Ethiopia, data were collected in 3 subcities of Addis Ababa. The study guide in Ethiopia focused on exploring the drivers and barriers to improved data quality and use practices at the point of health care delivery, the effect of capacity building on data quality and use, the effect of performance-monitoring teams (PMTs) in promoting the use of quality data for decision making, and the roles of leadership in improving data quality and use. In Ghana, the study was carried out in the Northern and Volta Regions. The administered interview guide included questions related to the development of the DDM intervention, barriers, and facilitators to its implementation both before and after its introduction, its perceived benefits, challenges faced since its introduction, and stakeholder perceptions of its sustainability. Additional questions to gauge support for data utilization from supervisors and perceived availability of resources to implement data-informed innovations were included for participating CHPS and subdistricts. In Mozambique, data were collected in the central provinces of Manica and Sofala.

In each country, researchers carried out qualitative analysis, incorporating “constant comparison” (i.e., deeply examining transcripts and comparing interpretations of text with existing findings as they emerge, thereby deriving themes to explore during analysis, and sets of codes for each theme).^38^ Then, analysts coded and reorganized data into coded segments that related to the themes.^39^^,^^40^ This resulted in a report from each country on the DDM implementation strategies, effectiveness determinants, and programming recommendations.

Working group members sent their reports to the working group lead and coordinator, who analyzed and collated findings according to constructs supplied by Leeman et al.’s implementation strategy classification schema and the determinants framework, the CFIR.^29^^,^^32^ Placing strategies into classes (via Leeman’s approach) supports systematic reporting of implementation research findings, connections to relevant theories, and identification of cross-country synthesis and strategy gaps. The CFIR provided a means to deductively conceptualize influencing constructs across 5 core domains of implementation effect (intervention characteristics, outer setting, inner setting, individuals, and implementation process). Meta-frameworks, such as the CFIR, are designed to go beyond assessing intervention effectiveness by identifying contextual influences that explain the heterogeneity of implementation success.^41^ All five domains (and 39 constructs/subconstructs) of CFIR were considered in the analysis, as considerable variability in drivers of implementation success and failure were expected across the 3 such heterogeneous settings in terms of geography, culture, and resources. Ultimately, 22 constructs/subconstructs were coded, with just 2 constructs mentioned across the 3 country projects and 8 noted by 2 country projects.

The working group lead coded the 3 reports and used memos to document thematic insight using qualitative software, QSR-Nvivo. Themes were based on further analysis of coded segments of the reports, including quotations obtained from report authors’ key informants. This was done in Microsoft Word. The teams shared the results with working group members who reviewed, added to, and ultimately confirmed the findings before finalizing convergence of opinion on key messages and programming recommendations.

### Ethical Considerations

Institutional review boards (IRBs) granted AHI-funded programs in each country ethical clearance and permission to conduct this research. The Data Use Partnership secured IRB approval from the Ethiopian Public Health Association (reference number EPHA/0G/074/20). Protocols for CHPS+ were approved by the Ghana Health Service (GHS) Ethics Review Committee (reference number GHSERC:04/01/17) and the Columbia University IRB (reference number AAAR0315). The Mozambique partnership obtained ethical approval from the Ministry of Health IRB (reference number IRB00002657) and the University of Washington (reference number STUDY00003926).

## COMPARING IMPLEMENTATION STRATEGIES FOR DDM INTERVENTIONS ACROSS COUNTRY SETTINGS

Implementation strategies have been defined as “methods or techniques used to enhance the adoption, implementation, and sustainability of a clinical program or practice.”^42^ They are, by definition, complex social interventions that are implemented across interpersonal, organization, and community contexts.^43^ Proctor et al. detail operational and conceptual prerequisites to studying implementation strategies, specifically naming, defining, and specifying the strategy.^30^ In Table 2, we name and define the implementation strategies adopted to foster data quality and use in each of the 3 partnership projects; we also list the actors, action, action targets, justification, temporality, and dose, as well as the implementation outcome for each action.

**TABLE 2.** Implementation Strategies to Foster Data Quality and Use in AHI-Partner Projects

**Ethiopia DUP was designed to scaffold onto the government's information revolution roadmap through leadership engagement, mentored and team-based capacity building, financial incentives, and investments in the eHealth architecture**					
**Actor**	**Action**	**Action Target and Justification**	**Temporality**	**Dose**	**Implementation Outcome**
Study staff	Leadership engagement	Work with MOH leadership at district, province, and national levels to define and bolster governance structures and define standards and responsibilities	Baseline and annually thereafter	Formal dissemination meetings at each level (1–2 hours)	Acceptability, sustainability
	Mentored supervision	Local universities provide individual HIS capacity building to health worker-led PMTs in target regions through the CBMP	At a minimum, quarterly mentored supervision	Supervision visits of PMTs (2–3 hours)	Adoption
	Investments in data use eHealth architecture	Financial support to upgrade technology for HIS as well as related learning platforms linked to the Connected Woreda strategy	Investments were made at the start of DUP to upgrade technologies		Penetration, sustainability
Study staff, facility staff	PMTs at health facility level	PMTs meet to review data, perform root cause analysis, LQAS, and define tailored action strategies for improvement	Monthly	Meetings monthly (2–3 hours)	Appropriateness, penetration
**Ghana's CHPS+ project approach provided investments into learning platforms and mentoring partnerships and reinforced data collection modalities (e-Tracker/Data dashboard)**					
**Actor**	**Action**	**Action Target and Justification**	**Temporality**	**Dose**	**Implementation Outcome**
Study staff, university partners	Linked systems learning districts and university-based learning process	Innovative approach to transfer system strengthening strategies from 4 districts to larger-scale implementation in additional regions (peer learning with financial incentives)	Peer learning introduced at baseline and followed up once at 6 months	Two trainings at baseline and 6 months for each systems learning district	Adoption, appropriateness
Study staff	Mentoring support	Peer learning operations prioritized including decision making and leadership	At minimum quarterly	Mentoring 4 times per year (1–2 hours)	Adoption
	Technologies to reinforce data collection and use (e-Tracker)	Data capture, analysis, and use will be prioritized through the use and expansion of the e-Tracker	Start-up and annual refresher trainings plus monthly use on-the-job	Training/refresher course (3 hours)	Adoption, penetration, sustainability
	Training to address skill gaps among health care team	Training to disseminate simple, low-cost, and rapid turnaround tools for impact monitoring to inform policy and practical decision making	Completed at baseline	2–3 hours	Acceptability, fidelity
**Mozambique IDEAs program was designed to adapt and expand a district-based audit and feedback approach and enhance capacity in research and targeted implementation studies linked to MNCH**					
**Actor**	**Action**	**Action Target and Justification**	**Temporality**	**Dose**	**Implementation Outcome**
Mozambique NIH	Service provision assessments and data quality audits	Periodic service readiness and data quality assessments of a rotating sample of randomly selected facilities in the study area	Conducted quarterly	1–2 days for each activity	Fidelity, penetration
Study staff, MOH managers	District performance review and enhancement meetings	Adaptation and expansion of the district performance and enhancement meetings into an iterative learning approach	Conducted twice annually	4–5 hours	Appropriateness, penetration
	Mentored supervision	Build and support implementation research, through mentoring visits that review action plans and use of facility support grants	Quarterly at a subsample of sites	2–3 hours	Adoption, implementation cost

Abbreviations: AHI, African Health Initiative; CBMP, capacity building and mentorship program; DUP, Data Use Partnership; HIS, health information system; IDEAs, Integrated District Evidence-to-Action program to improve maternal, newborn, and child health; LQAS, lot quality assurance sampling; MOH, Ministry of Health; PMT, performance-monitoring team.

Across the 3 projects, respondents cited mentoring and data-use engagement across health-system levels as common actions performed in support of DDM strategies. Mentoring was used to motivate health care personnel to work within the existing accountability structures. Each setting convened teams to review routine data, identify discrepancies, highlight best practices, and prioritize opportunities to strengthen health delivery systems; however, the actors engaged differed by project. The Mozambican project is the most embedded within the existing health system, with public actors engaged in each core activity. CHPS+ in Ghana invested in linking university scientists to field actors, to bridge the notorious research-practice divide. In Ethiopia, the Data Use Partnership focuses on data management and use at the health facility level as well as the provision of administrative support at the MOH, regional health bureaus, and district health offices to strengthen the health system’s use of routinely collected health information for decision making. Table 2 highlights data-use engagement modalities employed, including PMTs, bolstering linkages between universities and learning-based district teams, and district-based performance and enhancement review meetings.

Across the 3 projects, respondents cited mentoring and data-use engagement as actions that supported DDM strategies.

Specific changes in practices and management that resulted from the DDM strategies were different across settings. In Mozambique, the semiannual 4-day district performance review meetings (DPREMs) were adapted into prework and presentation phases. In the prework phase, district supervisors, with AHI team support, worked with facility health care staff to input, analyze, and develop accurate and compelling presentations of the data. This prework was individualized to facility staff needs and provided one-on-one mentoring to build their DDM skills, from data entry to analysis to dissemination. Prioritizing the individual skills building of team members before the joint presentation days made the entire event more supportive, engaging, and solution oriented.

*The intervention [DPREM] design and resources it allocates seem to address technical and logistical needs of the health system at the district and health facility level, particularly related to developing a positive learning climate and facilitating accountability regarding data quality and their usage to improve service deliveries by facilities and the maternal and child health program.* —MOH Medical Center Director, Mozambique

Both Ethiopia and Ghana invested in and supported mHealth technologies to reinforce data use. Ethiopia targeted investment in the government’s eHealth architecture priorities, whereas Ghana focused on facility-level mHealth tools to enhance timely health care data collection and use. The Mozambique project supported the collection and use of quarterly data assessments related to service provision capacity and routine data quality by the Mozambican National Institute of Health. While not an mHealth strategy, this effort effectively focused stakeholder engagement across levels of the health system to collect and analyze routinely collected data, systematically. The Mozambican National Institute of Health collected and presented the data, and results were shared at the provincial, district, and health facility levels to expand system-level accountability for data quality and use. In addition, linkages via WhatsApp bolstered communication across stakeholders.

## BARRIERS TO AND FACILITATORS OF EFFECTIVE IMPLEMENTATION OF DDM INTERVENTIONS

We used the CFIR to identify organizational-level determinants of successful or unsuccessful implementation of data-use strategies across the 3 project areas. The results of our analysis highlight barriers and facilitators of effective DDM interventions in the context of CFIR constructs (listed in Table 3).

**TABLE 3.** Consolidated Framework for Implementation Research Domains and Descriptions

**Domain**	**Short Description**	
**I. INTERVENTION CHARACTERISTICS**		
Intervention source	Perception of key stakeholders about whether the intervention is externally or internally developed.	
Evidence strength and quality	Stakeholders’ perceptions of the quality and validity of evidence supporting the belief that the intervention will have desired outcomes.	
Relative advantage	Stakeholders’ perception of the advantage of implementing the intervention versus an alternative solution.	
Adaptability	The degree to which an intervention can be adapted, tailored, refined, or reinvented to meet local needs.	
Trialability	The ability to test the intervention on a small scale in the organization and to be able to reverse course (undo implementation) if warranted.	
Complexity	Perceived difficulty of implementation reflected by duration, scope, radicalness, disruptiveness, centrality, intricacy, and number of steps required to implement.	
Design quality and packaging	Perceived excellence in how the intervention is bundled, presented, and assembled.	
Cost	Costs of the intervention and costs associated with implementing the intervention including investment, supply, and opportunity costs.	
**II. OUTER SETTING**		
Patient needs and resources	The extent to which patient needs, as well as barriers and facilitators to meet those needs, are accurately known and prioritized by the organization.	
Cosmopolitanism	The degree to which an organization is networked with other external organizations.	
Peer pressure	Mimetic or competitive pressure to implement an intervention; typically, because most other key peer or competing organizations have already implemented or are in a bid for a competitive edge.	
External policy and incentives	A broad construct that includes external strategies to spread interventions, including policy and regulations (governmental or other central entity), external mandates, recommendations and guidelines, pay-for-performance, collaboratives, and public or benchmark reporting.	
**III. INNER SETTING**		
Structural characteristics	The social architecture, age, maturity, and size of an organization.	
Networks and communications	The nature and quality of webs of social networks and the nature and quality of formal and informal communications within an organization.	
Culture	Norms, values, and basic assumptions of a given organization.	
Implementation climate	The absorptive capacity for change, shared receptivity of involved individuals to an intervention, and the extent to which use of that intervention will be rewarded, supported, and expected within their organization.	
Tension for change	The degree to which stakeholders perceive the current situation as intolerable or needing change.	
Compatibility	The degree of tangible fit between meaning and values attached to the intervention by involved individuals, how those align with individuals’ norms, values, and perceived risks and needs, and how the intervention fits with existing workflows and systems.	
Relative priority	Individuals’ shared perception of the importance of the implementation within the organization.	
Organizational incentives and rewards	Extrinsic incentives such as goal-sharing awards, performance reviews, promotions, and raises in salary, and less tangible incentives such as increased stature or respect.	
Goals and feedback	The degree to which goals are clearly communicated, acted upon, and fed back to staff, and alignment of that feedback with goals.	
Learning climate	A climate in which: (1) leaders express their own fallibility and need for team members’ assistance and input; (2) team members feel that they are essential, valued, and knowledgeable partners in the change process; (3) individuals feel psychologically safe to try new methods; and d) there is sufficient time and space for reflective thinking and evaluation.	
Readiness for implementation	Tangible and immediate indicators of organizational commitment to its decision to implement an intervention.	
Leadership engagement	Commitment, involvement, and accountability of leaders and managers with the implementation.	
Available resources	The level of resources dedicated for implementation and ongoing operations, including money, training, education, physical space, and time.	
Access to knowledge and information	Ease of access to digestible information and knowledge about the intervention and how to incorporate it into work tasks.	
**IV. CHARACTERISTICS OF INDIVIDUALS**		
Knowledge and beliefs about the intervention	Individuals’ attitudes toward and value placed on the intervention as well as familiarity with facts, truths, and principles related to the intervention.	
Self-efficacy	Individual belief in their own capabilities to execute courses of action to achieve implementation goals.	
Individual stage of change	Characterization of the phase an individual is in, as he or she progresses toward skilled, enthusiastic, and sustained use of the intervention.	
Individual identification with organization	A broad construct related to how individuals perceive the organization, and their relationship and degree of commitment with that organization.	
Other personal attributes	A broad construct to include other personal traits such as tolerance of ambiguity, intellectual ability, motivation, values, competence, capacity, and learning style.	
**V. IMPLEMENTATION PROCESS**		
Planning	The degree to which a scheme or method of behavior and tasks for implementing an intervention are developed in advance, and the quality of those schemes or methods.	
Engagement	Attracting and involving appropriate individuals in the implementation and use of the intervention through a combined strategy of social marketing, education, role modeling, training, and other similar activities.	
Opinion Leaders	Individuals in an organization who have formal or informal influence on the attitudes and beliefs of their colleagues with respect to implementing the intervention.	
Formally appointed internal implementation leaders	Individuals from within the organization who have been formally appointed with responsibility for implementing an intervention as coordinator, project manager, team leader, or another similar role.	
Champions	Individuals who dedicate themselves to supporting, marketing, and “driving through” an [implementation], overcoming indifference or resistance that the intervention may provoke in an organization.	
External change agents	Individuals who are affiliated with an outside entity who formally influence or facilitate intervention decisions in a desirable direction.	
Executing	Carrying out or accomplishing the implementation according to plan.	
Reflecting and evaluating	Quantitative and qualitative feedback about the progress and quality of implementation accompanied with regular personal and team debriefing about progress and experience.	

### Intervention Characteristics

Intervention characteristics, the first major domain of the CFIR, relate to details on how the intervention was implemented in or across a particular organization. Project respondents described their perceptions of DDM strategies and the degree to which various characteristics of those strategies impeded or facilitated implementation success.

#### Evidence Strength and Quality

In Ethiopia, DDM interventions were seen as more effective in promoting data use to track health care worker performance and guide decision making when compared to previous approaches. Stakeholders reported that the quality of routine data improved, and data were increasingly used to measure implementation across the system, which was the desired outcome.

*Generally, what we do (now) is highly related to data; starting with forecasting of drugs, estimating budget. Similar to this, across the different departments, decisions are made based on data. For instance, if there are stillbirths which are increasing in the health facility, we ask for justification. When the top ten diseases are listed, it is based on data that we do ABC analysis, vein analysis, and that essential drug lists are also defined by looking at which diseases are common, which is also based on data; so, we use the data for many activities*. —MOH health center medical director, Ethiopia

In Mozambique, the DPREM structure allows for review of routine data and of innovations and practices leading to service delivery improvements. Held in district capitals, DPREMs are conducted every 6 months, bringing together district supervisors, facility-level managers, and typically some primary health care workers, to review and discuss facility-level data in an open, collegial format. Additionally, Ethiopian health care workers appreciated the audit-and-feedback structure of DPREMs and mentored supervision follow-up visits. Over time, they were able to see that their review of data could be used to guide discussions, and the time together could generate practical solutions that resulted in improved health services and uptake by women.

*There is competition among the health facilities, which contributes to improving data quantity and quality. These meetings are also a reminder to the districts about what is going on and what needs to be done.* —MOH district supervisor, Mozambique

*For example, (through [Integrated District Evidence to Action], we introduced family planning services in other sectors of our health facility. This integration of family planning services increased our numbers of women using family planning methods, but also improved the perception of our services to community members. They can see that we provide a range of services for our population no matter what unit you enter.* —MOH MNCH nurse, Mozambique

In Mozambique, the DPREM structure allows for review of routine data and of new innovations and practices leading to service delivery improvements.

#### Relative Advantage

In Ethiopia, consistent capacity building via didactic training and on-the-job mentorship has been appropriately designed to meet health care workers’ skills-building needs. Thus, health facilities have moved fluidly from emerging to candidate and model levels, per the objectives of the country’s Connected Woreda strategy.

*Mentorship has a significant contribution in improving data quality and strengthening the recording system. The majority of the health institutions shifted from “emerging” to “candidate’ and from “candidate” to the “model” level. This is because we provided mentorship closely through facilitating knowledge and experience sharing. The mentorship was conducted every month and all the necessary corrective measures were taken on the spot and feedback was given to the concerned stakeholders. All the 36 health institutions in the 3 subcities where a strong mentorship has been done are in good status with regard to data quality.* —Regional health bureau representative, Ethiopia

The baseline HMIS system in Ghana required tallying information across multiple registers at the facility and community levels in CHPS zones before summarizing district-level reporting forms. Subsequently, the District Health Information Management System (DHIMS) web-based software and e-Tracker interface provided easier visibility of routine data across levels, which reduced errors. This, in turn, resulted in the perception among health care workers and managers that data were more trustworthy and therefore more useful for decision making.

*People doing this manually can lead to errors, transposition errors, calculation errors and all that, so we created an electronic platform…using the [District Health Information System] software.* —GHS, national level, Ghana

According to national-level stakeholders in Ghana, CHPS+-supported DDM interventions were designed to be a better solution to the existing system and were intended to improve data reporting timeliness, increase facility-level ownership, and support the use of DDM.

*…to resolve that [lack of timeliness in data reporting and ownership of data at the facility level] we decided that as part of the intervention for data for decision making, facilities should be empowered to do the entry on the electronic system, it reduced [health care workers’] workload and improves the quality, and also frees the district teams to do more quality checks…they can provide oversight over the quality.* —GHS, national level, Ghana

One advantage of Ghana’s e-Tracker is its ability to support default tracing, which allowed community health officers to plan follow-up home visits with clients who missed appointments or children who missed scheduled vaccinations. Before the introduction of the e-Tracker, health care workers manually went through registers to search for defaulters, which they did not do with frequency due to time constraints.

*One other thing too is, now with CHPS+ coming on board, we had e-tracker. It came with challenges at the beginning, but now they are able to use the e-tracker to trace their defaulters, their defaulters in [Expanded Program on Immunization] coverages. So hence once they capture the clients, the individual clients and the client’s default, they know that this client has defaulted so they follow up. So that has brought about improvement so we’ve seen improvement in for instance Penta 3, yes, which is used as proxy for [Expanded Program on Immunization] coverage so that is what is in addition. —*GHS, health information officer, Ghana

#### Adaptability

Sites across the Mozambique Integrated District Evidence-to-Action program to improve maternal, newborn, and child health (IDEAs) added the use of weekly WhatsApp feedback between district supervisors and facility managers. Through this approach, district supervisors asked questions and highlighted data discrepancies with each facility in their region in a more timely manner. This adaptation to more frequent check-ins made communication around primary data source correction more routine and open, leading to faster learning and improvement.

*Data are also shared through WhatsApp groups, which are divided into a group of MCH nurses and MCH facility managers, MCH district managers, and a broad group of district health staff. I once had doubts about some contents. I shared those doubts in the group and I received help.—*FGD participant, nurse, Sofala Province, Mozambique

#### Trialability

One of the more successful approaches of Ethiopia’s Data Use Partnership was allowing health care teams at the facility to work through challenges in routine data together with university partners during joint consultative sessions. This development led to more confidence and engagement once the health care team returned to the facility and continued the process independently.

*The data quality improved significantly when we involved owners of the data in the problem identification and to resolve it. What we did recently is that we invited (health care team members) from a specific health facility who thoroughly discuss and assess their own problem during a consultative session organized by us, which was found to be fruitful. While they are discussing about their problems, we (university team) comment or propose ideas for their considerations. Once they are back to their facilities, we follow their progress in addressing the identified challenges and I think this is an innovative idea. —*Addis Ababa University member, Ethiopia

Ethiopia’s Data Use Partnership allowed health care facility teams to work through challenges in routine data together with university partners during joint consultative sessions.

#### Complexity/Design Quality and Packaging

In Ghana, some core activities of the intervention required internet access, which was often unavailable or spotty. When such connectivity issues stymied implementation, staff users' acceptance of these internet-dependent activities was diminished.

In Ethiopia, challenges included shortages of checklists and associated data collection/compilation tools, internet connectivity issues with the HMIS digital system, and gaps in understanding data and indicators. Structured, regular supportive supervision visits, guided by a checklist, were key to sustained and systematic improvements in both data quality and use.

*What we do to prevent data quality problems is, first we give on-job training and second we also conduct supervision. During the supervision, verbal and written feedback is provided on issues needing improvement. —*Study team – disease prevention and health promotion coordinator, Ethiopia

### Outer Setting

The second domain of the CFIR, outer setting, typically includes the economic, political, and social context within which the organization resides.

#### Cosmopolitanism

In Ghana, stakeholders of the CHPS+ project reported that benefits arose from having a broad range of funders and partners contributing to the MOH’s DDM strategy including the U.S. Agency for International Development, Gavi, Vaccine Alliance, World Bank, Korean International Cooperation Agency, Samsung, Global Fund, and Doris Duke Charitable Foundation.

#### External Policy and Incentives

Implementation of health insurance bolstered Ethiopia’s data quality efforts because health facilities must provide complete service information about services rendered to be reimbursed.

*Health insurance systems became one opportunity for use to ensure data quality because when patients come to our health institute for care, they need to have full information including full medication history for insurance to cover the costs. —*MOH maternal and child health coordinator, Ethiopia

A major barrier expressed by Ethiopian study informants was inadequate staffing to handle the amount of data management work at the health facility level. Most health facilities in the country suffer health information technician (HIT) shortages due to a government initiative encouraging HITs to upgrade their diploma-level training to Bachelor’s degrees. This initiative allows HITs to keep their facility-level salary and position while attending college to complete the 2–3-year training program, which in turn limits health facilities’ ability to hire replacement staff while the HITs are away.

*We have only 1 [health management information system] officer now because the other went to higher education and we can’t recruit a replacement. We have no option except waiting until she completes her education but this has influence on data-use work, which means the data-use activities will be handled by 1 expert. —*MOH health center medical director, Ethiopia

An important GHS initiative is to promote the use of information and communication technology to enhance service delivery. As an embedded component of this initiative, the e-Tracker provides a platform to link and manage emerging technologies and applications.

*…Provide an [information and communications technology] platform for implementing, securing and managing emerging technologies and applications that support the health care delivery system. —*MOH national level, Ghana

In Ghana, CHPS+ provided an opportunity to scale the project to additional regions. GHS wisely leveraged support from international organizations and partners to scale this work and support the achievement of its goals to improve data quality and use across all levels of the health system.

*We tell people, we are very clear in our minds what we want to do, we just don’t have the money to do it, so we look around … we leverage on all the resources we can get. —*GHS, national level, Ghana

External policies that partly informed DDM elements of CHPS+ included Ghana’s e-health Policy, the Data Protection Act, and individual policies specific to disease programs that adopted data strengthening interventions.

In Mozambique, the national-level HMIS (SISMA/DHIS2) dictates which data to collect; it also requires district managers to regulate such collection before sending the data to provincial/national levels. The IDEAs project targeted strengthening of district management, ensuring that it was in line with MOH priorities.

### Inner Setting

Inner setting, the third major domain of the CFIR, includes features of structural, political, and cultural contexts through which the implementation process proceeds.

#### Culture

Although data quality and use improvements were appreciated in the Ethiopian context, informants emphasized that more is needed to foster a culture of information use at health facilities.

*I think data-use culture is poor and we don’t know exactly how they (health workers) use data for decision making since we don’t participate in management (PMT) meeting. We see that case team coordinators and core process owners meet with health center management and we don’t have knowledge whether they use it as input for management decisions. We submit data to management on monthly basis and I don’t know if they use it or not and it is better if they can engage us in the management meeting so we can be resources for them in providing the necessary data. —*Study team HIT, Ethiopia

Although data quality and use improvements were appreciated in the Ethiopian context, informants emphasized that more is needed to foster a culture of information use at health facilities.

#### Implementation Climate

**Compatibility**. In Ethiopia, respondents reported that the presence of duplicative data collection registers at health facilities, some introduced by vertical programs (such as for HIV), posed a challenge to ensuring the completeness and consistency of collected data. This issue also burdened health care workers by making it challenging to keep all registers up to date, thereby lessening the data’s perceived value and use.

*There are many registration books to fill. For instance, in the Operation triple A report, an HIV reporting template introduced by CDC, we usually find the mismatch between weekly and monthly report. When we tried to do it again, we find that there are many different registrations that are used as the source document…there is the main registration itself, there is risk assessment registration at outpatient department and there is provider-initiated testing and counseling. —*MOH FGD participant, Ethiopia

**Relative Priority.** In Ghana, over time, skills-building training and associated tools (e-Tracker and DHIMS dashboard) to strengthen data in the CHPS+ project areas resulted in improvements in stakeholders’ perceptions of the importance of data quality and its use at the facility and district levels. Because many of these approaches are novel to health care workers and managers, their perceived advantage over the status quo emerges over time.

*So, I feel the frontline staff are now beginning to appreciate data. They are now understanding the essence of data. So now, because when the e-tracker came on board and then we trained them. We also took them through CHPS+ training, so now they enter their own data at the field. So, they know that they are not performing, even though we provide monthly feedback to them as well. But as they are entering and they are going through the list, they know that no, we are not performing in this area. We have to speed up or we have to move to or conduct a mop up. So, I’ll say that, they are beginning to appreciate the data much more. —*District health information officer, GHS

*Before then I never knew there was something there like e-Tracker, there were something like dashboard that you can use it to monitor your performance, so it has really helped us a lot. —*Public health nurse, GHS

In Ghana, skills-building training and associated tools to strengthen data in the CHPS+ project areas improved stakeholders’ perceptions of the importance of data quality and its use at the facility and district levels.

**Organizational Incentives and Rewards.** In Mozambique, district supervisors generated enthusiasm for the IDEAs DPREM activities by offering organizational incentives and publicly acknowledging achievement and/or improvement. In 1 district, high-performing facility staff received prizes (e.g., medical supplies or equipment) for their facilities, and lower-performing facilities with demonstrated improvement were publicly recognized (e.g., as “most improved”) to encourage the health care team’s continued progress.

**Learning Climate.** Facility-level health care workers and HITs in Ethiopia reported technical capacity gaps, specifically in disease reporting, due to limited staff training on the national classification of diseases. Team members reported that feeling knowledgeable was important to engage as effective members of change processes.

#### Readiness for Implementation

**Leadership Engagement.** Multiple participants in the Ethiopian project mentioned challenges in data-use capacity at the leadership level. Regional leaders also expressed dismay at their inadequate training to effectively support the HMIS, thus negatively impacting its success.

*As to me it is good to start working towards the concept of data use at leadership level because the majority of people who are in the leadership role don’t have adequate knowledge towards data use including me. Therefore, in the future, training should be provided to all concerned bodies starting from the leaders about data use and evidence-based decision making. Data is important for decisions, budget allocation, and political decision so knowledge of data use is crucial for leaders and all other concerned stakeholders. Thus, training will be organized and given to all leaders and other stakeholders to utilize data properly for decision making and this should be given due emphasis in the future. —*MOH Addis Ababa regional health bureau, Ethiopia

CHPS+ data quality and use intervention results analysis in Ghana also highlighted contextual- and system-level factors that enhanced or inhibited the adoption of novel interventions. Here too, leadership was found to be critically important. A review of qualitative systems appraisal (QSA) data from the CHPS+ project demonstrated that Central Tongu, one of the System Learning Districts, made significant improvements in data utilization for system performance and improvement. Consistently, both health care workers and district managers from Central Tongu attested to their improved capacity to use data for service innovation and data reporting. They saw these improvements as arising due to the influence of leadership and the availability of tablets installed with the offline version of the DHIS. These tablets enabled facility staff to directly enter their reports, rather than tally numbers by hand and deliver them to subdistrict or district supervisors. In sites where leadership and enabling technology are present, and funds exist for resource upgrades and training, truly transformational change can occur.

In Mozambique, leadership provided considerable support, especially as the project was deeply embedded within the actual DDM interventions, specifically at the district and provincial levels. Respondents also expressed considerable appreciation for the support and engagement of the AHI partner.

*Yes, yes, it is, yes, yes. The “tecnicos” (mid-level providers) who actually go for Data Review and Enhancement Meetings, with the health facility colleagues, then receive follow on mentored supervision with the district medical chief, the provincial [maternal, newborn, and child health] supervisor, and the provincial statistics lead. So, it really is the true people who are prepared to work on these issues with the (health facility) team. —*MOH maternal, newborn, and child health provincial supervisor, Mozambique

**Available Resources.** IDI and FGD respondents from the Ethiopia project cited considerable challenges to implementation related to the underdeveloped health information system infrastructure. Challenges included limited space, furniture, computers, and shelving that compromised the organization and use of stored data. Insufficient working infrastructure also created concerns of potential vulnerabilities to data security and confidentiality.

Ethiopian respondents also saw the instability of the DHIS2 due to connectivity issues and other related problems (e.g., slow processing times to generate analysis) as a major challenge in the project, further frustrating health care workers who depend on this system for their daily data-use work. Because the DHIS2 system is not reliable, higher-level bodies who need data tend to repeatedly ask health facilities to send data directly rather than accessing the data from the DHIS2 system.

*The other challenge is on the medical record unit; there is interruption of network (internet), workload in the archive unit and the rooms are confined, there is space problem, privacy and confidentiality problem since many people have access to the computers. —*MOH Addis Ababa regional health bureau, Ethiopia

*Another challenge was recurrent interruptions of district health information system version 2 (DHIS2), it was down for long time and we couldn’t even analyze 10 top diseases, we did it manually. There are also problems of getting help when we need maintenance service. —*MOH health center medical director, Ethiopia

*Currently, a very important but difficult thing is the DHIS2 system. However; we are asked by different bodies; for example, subcity is asking the same data in 5 ways, which means they don’t see DHIS-2 report. And, if someone needs data, he/she calls us instead of getting the data from DHIS2. In this modern time, we are behind technology; even we cannot exchange report from outpatient department through computer. —*MOH FGD participant, Ethiopia

In Ghana, the procurement of tablets for all facilities in the implementation districts allowed staff to enter monthly data directly into the DHIMS instead of bringing it to the district level for entry, which improved reporting completeness and timeliness. An additional benefit of tablet procurement was staff’s use of them in conducting research for community health talks. At the outset of the project, considerable investments were made to support the CHPS+ data infrastructure including tablets, data tools (e-Tracker and dashboards), and supplies (registries), as well as staff capacity building via training. Respondents from some districts recognized that these investments improved timely facility support when care quality, data gaps, or inconsistencies arose.

*With the e-Tracker platform at the end of the month, the service data is also entered into DHIMS too. So now CHPS+ compounds will not now bring their reports to the district level again. Their capacity has been built.” —*CHPS+ coordinator, GHS

*It has strengthened the community programs. Recently we just had an invite from a nearby community Adeagpe, they invited us to deliver health talk during their Student Union Day, because of the CHPS+ program we had our motorbikes filled with fuel, we had gadgets, laptops, tablets we were able to do online research to be prepared for the health talk.” —*Community health officer, GHS

*During our situational analysis where… the monthly data that we’ve been collecting from the zones… we saw traditional birth attendant deliveries increasing, those are the things that we quickly strategized and then sent midwives to those places. —*CHPS+ coordinator, GHS

Insufficient workforce staffing was identified as a key barrier to successful IDEAs implementation in Mozambique.

*The problems of lack of personnel at health facilities is very serious. Lack of personnel creates work overload to the few people who are there, and this leads to low data quality. If the project has the means, it should help by hiring more MNCH personnel. —*MOH district supervisor, Mozambique

**Access to Knowledge and Information.** In Ghana, district health management teams in CHPS+ project areas highlighted specific examples of data quality and use improvement through increased access to digestible information and improved knowledge of how to incorporate data into work decisions. In Mozambique, not all facility-level staff attend DPREMs, so their ability to improve their data-use skills depended upon others who participated. However, limited staffing and insufficient time reduced the “share back” of DPREM learnings. To deal with this, some districts created a rotating attendance schedule so all health staff could periodically attend the DPREM, but that approach slowed learning and impeded the sense of teamwork across the district facilities.

### Characteristics of Individuals

The fourth major domain of the CFIR is the individuals involved in the intervention’s implementation. Individuals have agency; they make choices and can wield power and influence on others with expected or unexpected consequences for implementation. Individuals are also carriers of cultural, organizational, professional, and individual mindsets, norms, interests, and affiliations.

#### Self-Efficacy

Community health officers in Ghana received considerable support through the CHPS+ data quality and use efforts. As a result, they have been able to contribute more meaningfully to ensuring data quality and timeliness of reporting (specifically in antenatal care and Expanded Program for Immunization), and they infuse their reporting to the facility and district health departments with their inherently better understanding of community characteristics and priorities.

In Mozambique, health care providers noted improvements in their ability to understand, collect, and use data because of the IDEAs project.

*Practically, I will say, before there were certain indicators I had pretty low before compared to now. At least now I know I can reach the goals, and I know which indicators are the most important. I know that fourth antenatal care visit is important and why. I know it is important for pregnant women to come in early, so I tell them to come and don’t send them away. I know it is good for their partners to come, so they can be part of the decisions. I know the (data and its use) is better now than it was before. —*MOH facility MNCH nurse, Mozambique

In Mozambique, health care providers noted improvements in their ability to understand, collect, and use data because of the IDEAs project.

#### Individual Stage of Change

In Ethiopia, a major barrier mentioned repeatedly by the study participants was health care workers’ unfavorable attitudes/perceptions about and commitment to data quality and use as part of their job. Health care workers focused more on providing care for patients and less so on properly recording, reporting, and using health data.

*There is an attitude problem [among health workers]. Sometimes the performance monitoring team didn’t utilize data properly. Even some of the health professionals didn’t fill the patient profile as per the standard and this is due to attitude problem and negligence.* —MOH Addis Ababa Regional Health Bureau

### Implementation Process

Implementation process, the fifth CFIR domain, is the change process that occurs to facilitate the use (by individuals or the organization) of the intervention as it was designed/intended.

#### Engagement

**Formally Appointed Internal Implementation Leaders.** Study team members in Ethiopia highlighted the exceptional role of the PMT (comprised of facility staff) in promoting data quality in the Ethiopian context. PMT members review health facility data monthly for consistency, completeness, and other parameters and validate its quality before it is inputted into the HMIS and used for further analysis and decision making.

*Reports are being reviewed by the PMT members. We also conduct a gap analysis including keeping all responsible individuals accountable for identified gap; this is one of the factors that helped to improve data quality in our facility. —*Study team MNCH coordinator, Ethiopia

Some districts in Sofala Province, Mozambique, enlisted health care workers from high-performing sites to attend supervision of lower-performing sites to share best practices. This peer-to-peer support was designed to promote more psychological safety in the mentored supervision process.

**External Change Agents/Opinion Leaders.** In Ethiopia, some respondents mentioned the Addis Ababa University-supported “KAP-stone” process. MOH facility staff in Ethiopia explained that regular, supportive supervision visits from higher-level experts helped solve data quality challenges. Their formal and informal influence coupled with the use of a checklist helped sustain data quality improvement, according to MOH informants.

*We implement KAP-stone projects in 3 subcities: Yeka, Akaki-Kality and Lideta where health facilities identify problems on data quality and information use and intervene to solve them and we have 3 teams composed of people from subcity level and responsible to implement and do research on KAP-stone project intervention. —*Addis Ababa University member, Ethiopia

*What we do to prevent data quality problem is, first we give on-job training and second we also conduct supervision. During supervision, verbal and written feedback is provided on issues needing improvement. —*Study team disease prevention and health promotion coordinator, Ethiopia

#### Executing

In Ghana, because similar projects have been tested and scaled in other regions, no pilot studies were conducted before implementation. Rather, a stepped implementation approach was used to introduce the e-tracker. Implementers ensured the necessary inputs (training, resources, and tools) were put into place and that lessons learned were continuously gathered from the study sites. Standard operating procedures were updated as adjustments were made. Thus, implementation was done in tandem with reflection on and evaluation of progress to incorporate quality and efficiency updates.

*We already have a thinking about how we want to improve things, so we are using people’s [programs] to achieve it … we have a clear agenda … we don’t use the word pilot, we have a clear agenda. We do the step ladder implementation; it is just that we don’t have the money … all the processes in the [CHPS+] Service Learning Districts are being done everywhere else. —*GHS, national level

Introduction of the e-tracker was described differently at the subnational level in Ghana. Limited health personnel, incomplete training coverage, and high turnover of staff, as well as perceptions of increased workload, diminished health care worker enthusiasm for the e-tracker over time.

*Well, the situation now, like I said…, more or less, I would say that the momentum is not as it used to be like before and the reason being that, number 1, those that were trained most of them had left, and so as they left, the incoming new ones were not trained on the job or they were not brought into the picture on how to use it, then that means that those who were trained and were taught on how to use it, as they leave the scene then a gap is being created. —*DHMT member, GHS

### Reflecting and Evaluating

Another strategy applied to promote data quality and use in Ethiopia was PMTs’ routine use of the Lot Quality Assurance Sampling approach. After collecting departmental data, HITs presented organized data to the PMT, which then randomly selected sample data from a program (e.g., TB or immunization) to review in-depth for accuracy and consistency and cross-check the sample with primary source data. This technique has been integrated into the routine work of health facilities supported by the Data Use Partnership to routinely address data quality issues.

*We sit for a PMT meeting and we conduct lot quality assurance sampling. When we usually sit for the PMT meeting, we sit having the report they submitted, the report I entered in the DHIS because I might also make a mistake while entering the data; most of the time they submit the report from day 21–22 of the reporting month and I usually finalize my report on 23rd –24th. Then, if [Expanded Program on Immunization] room is selected for the lot quality assurance sampling, we got to the department, and we check the source data including the tally sheet, my report. This is how we try to check the quality and solve the problem. —*MOH HIT, Ethiopia

## DISCUSSION

We applied a CFIR-guided lens across the 3 AHI-supported projects—in Ethiopia, Ghana, and Mozambique— to highlight differing drivers of DDM implementation success and failure in each country. Regarding characteristics of the respective interventions, we noted benefits when DDM strategies leveraged existing HMIS infrastructure and processes, which led to performance improvements in real time and were, therefore, deemed relatively better than “business as usual.” Similarly, Soti et al. report that immediate, tangible health care worker experiences of benefiting from the usability of an electronic mobile system (to assist in collecting malaria diagnosis and patient data) led to enthusiastic use of higher quality data—improved care and favorable perceptions of DDM acceptability and feasibility in rural settings.^44^

We noted benefits when DDM strategies leveraged existing HMIS infrastructure and processes, which led to performance improvements in real time and were, therefore, deemed relatively better than “business as usual.”

In Ethiopia, such beneficial effects were enhanced when implementers reported being able to try out different data-use approaches, work through challenges with mentors, learn about, and then adopt the DDM strategy gradually as health care worker confidence developed. Mozambican participants emphasized the importance of the adaptability of the intervention to implementation success, pointing out that health care worker and supervisor adaptations of the DDM strategies proved key to improvements in data quality and circulation of supervision feedback in project-affiliated sites. Insofar as these DDM strategies emphasize adaptable and iterative learning and capacity development, they help fill knowledge gaps about what works to improve data and use of routine data that were identified by a global scoping review on this subject that could point to only 3 interventions that focused on improving health care worker self-assessment and feedback in routine health information systems.^4^^,^^45^^–^^47^ In all countries, benefits arose from aligning DDM strategies with national policy processes and working effectively within the wider political climates. These constructs of the CFIR’s outer setting domain reinforced and incentivized DDM uptake and performance, above and beyond the inputs and supports offered by the projects. Comparable lessons emerged from the WHO’s Rapid Access Expansion Program, which used “data quality assurance” as a core strategy to strengthen national integrated community case management of childhood illness programs in 5 sub-Saharan African countries. This program reported that the ability of all health systems to benefit from data quality assurance requires policy supports that avail resources for supervision, training, and health care worker team initiatives to act on recommendations.^48^

The use of CFIR constructs within the inner setting domain yielded rich lessons, specifically those focusing on implementation climate and organizational readiness for implementing change related to DDM. In Ethiopia and Mozambique, AHI projects enhanced the climate for integrating DDM in primary health care settings by offering facilities incentives for exemplary use of the intervention. In Ghana, introducing new technology elevated stakeholders’ perceptions of the importance of DDM beyond the practical benefits of the e-Tracker, which district-level informants associated with better access to information and more informed decision making. In all projects, participants observed that benefits emerged from implementers’ engagement of facility, local, and, often, national government leaders in adapting and improving current HMIS practices and procedures to better reinforce effective DDM. Although in line with current emphases placed on establishing “learning health systems” in implementation science, our observations are novel in the canon on the determinants of data quality and use in LMIC systems, which has focused on intervention characteristics and processes.^49^^,^^50^ Previous studies focus on organizational environments and ways that overarching strategic management practices can nurture an underlying absorptive capacity for new information, learning capacities, and readiness to adapt; however, they are restricted to high-income country health systems.^51^^–^^55^ Future studies on this topic in LMICs should build on these findings and help fill this knowledge gap.

All projects noted challenges arising during the early stages of introducing DDM strategy activities. Stakeholders reported consistent lapses in organizational readiness for the intervention, specifically regarding interruptions in internet access, which contributed to HMIS system instability in countries, supply chain bottlenecks, health care worker shortages, and inadequate staffing arrangements. In Ethiopia and Mozambique, participants lamented persistent capacity gaps among health care workers who did not have the opportunity to directly engage with the AHI programs. In Ghana, participants noted that lapses in DDM strategy implementation were often due to the unavailability of funds for health care workers to enact data-driven solutions. Indeed, these findings reflect a wider consensus on the importance of underlying systems functionality for the success of DDM strategies. Program implementers should consider these conditions carefully and select the DDM approaches most likely to benefit from existing sources of support and to succeed despite prevailing deficiencies in the health system.

Beyond the inner setting, participants from all countries associated specific implementation processes with DDM success. Salient among these was the use of peer exchanges between workers from high-performing and average or low-performing jurisdictions; skills-building collaborations between health care providers and academic groups; and promotion of mentoring, reflection, and evaluation activities onsite by primary care teams. These findings echo lessons from more rigorous studies on “quality improvement collaboratives” and “mentoring and enhanced supervision” strategies, which demonstrate the feasibility and effectiveness of approaches to (1) horizontally scale data-driven quality improvement and (2) institutionalize capabilities to that effect in district health systems in LMICs.^56^^–^^58^

Interventions that were successful included peer exchanges between workers from high-performing and low-performing jurisdictions; skills-building collaborations between health care providers and academic groups; and promotion of mentoring, reflection, and evaluation activities onsite by primary care teams.

Challenges related to deploying digital technologies were noted in both Ghana and Ethiopia where they were a core DDM implementation strategy. For instance, the Ethiopian government made considerable initial investments to upgrade adjunctive learning support platforms, intended to bolster the Connected Woreda Strategy; however, both internet connectivity and suboptimal capacity in digital technology among medical registration unit workers and HITs have hindered the quality and use of data at the point of care. Training of facility staff involved in data collection, entry, and analysis was reported as incomplete and inconsistent at the point of care, which was reported as a barrier to the successful implementation of the Connected Woreda objectives.

The Ghana project, in support of CHPS+, contributed support to the introduction of the e-Tracker via android tablets in MNCH services. Rather than piloting the tablets before introduction, Ghana designed a “step-ladder” approach, where implementation began and, hypothetically, lessons learned were continuously incorporated into planning. The downside of this approach was that critical adjustments were not made before scaling and were instead made over time. This approach of real-time micro-adjustments heightened prospects of diminished health care worker acceptability over time. Although e-Tracker was anticipated to decrease the workload of primary health care and community health workers, many reported an increase in workload. Due to insufficient training using the tablet-based e-Tracker, many health care workers continued using paper-based registries and then were forced to spend additional time on data entry. This effort was further stymied by inadequate staffing and staff attrition. Training did not cover all staff members, peer training at the facility often did not occur, and refresher training was not provided. While efforts in both countries were reported as successful by respondents in both countries, implementation challenges persisted, mainly due to insufficient investment in staff development. Recommendations from respondents were consistent across the projects. Ensuring ongoing training for health care teams was paramount. Respondents also recommended using innovative modalities, such as self-learning digital tools, and developing the capacity of facility leadership, through training on health information system basics and effective DDM strategies, as part of standard onboarding when (inevitable) leadership transitions occur.

Insights that emerged from this cross-country comparison of qualitative evidence from similar projects in the 3 countries supported the development of a common theory of change to strengthen data quality and use in LMIC settings (Figure). Our theory of change identifies 3 common areas of necessary investment: data systems and practices, human capacity, and data visualization. In this model, introducing specific data systems and data practices both motivated health care workers and made them more accountable to improve their routine health data. Subsequent investments in health care worker capacity for data use are required, including standardized DDM training packages. By fostering capacity, motivation, and stronger accountability systems, such investments can increase demand for high-quality data in accessible, ready-to-use formats among both health care workers and managers.

**FIGURE.**
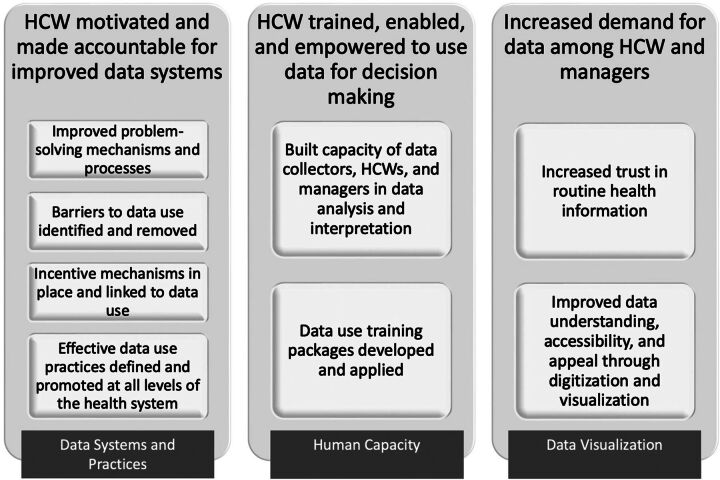
African Health Initiative Theory of Change to Strengthen Data Quality and Use in Low- and Middle-Income Settings Abbreviation: HCW, health care worker.

Our theory identifies 3 common areas of necessary investment: data systems and practices, human capacity, and data visualization.

The best practice identified across the 3 projects was to embed these efforts into the existing health infrastructure to optimize DDM adoption, reach, and effectiveness. Overall, this approach strengthened communication and prioritized data collection, analysis, and use, thus fostering accountability across health service levels. As DDM is viewed as an internal priority, quality and use are owned and employed by policy makers and managers, and resources and innovation are freed up to meet the increasing demand for data.

## CONCLUSION

Despite differences in programming context and approaches, this analysis underscores lessons that may help optimize future DDM projects for high performance in LMIC settings. Facilitating opportunities for team-based capacity building that is focused on data interpretation and sharing and individual mentorship led to effective DDM programing in all AHI partnerships. External policies and associated incentives bolstered this effect, but on occasion led to unintended consequences, for example, when motivational schemes that permitted health care staff to pursue further education without ensuring their replacements led to breakdowns in DDM practices. Leadership engagement and availability of resources to act on recommendations, respond to capacity-building needs, and facilitate collaborations between peers and within hierarchies across local health systems were crucial to DDM endeavors, as were implementation strategies that encouraged adaptation and opportunities for iterative on-the-job learning. DDM interventions grounded in these principles and practices are likely to succeed and help drive wider health systems strengthening in the countries where they are conducted.

**AHI Partnership Collaborative for Data Use for Decision Making Authors:** Sarah Gimbel (Department of Child, Family & Population Health Nursing, University of Washington, Seattle, WA, USA; Department of Global Health, University of Washington, Seattle, WA, USA); Colin Baynes (Department of Global Health, University of Washington, Seattle, WA, USA); Hibret Alemu Tilahun (Ethiopia Data Use Partnership, John Snow International, Addis Ababa, Ethiopia); Lisa Hirschhorn (Feinberg School of Medicine, Northwestern University, Chicago, IL, USA); Celso Inguane (Department of Global Health, University of Washington, Seattle, WA, USA); Pearl Kyei (Regional Institute for Population Studies, University of Ghana, Accra, Ghana); Biruk Abate (Ministry of Health, Addis Ababa, Ethiopia); Hiwot Belay (Ethiopia Data Use Partnership, John Snow International, Addis Ababa, Ethiopia); Abebaw Gebeyehu (Ethiopia Data Use Partnership, John Snow International, Addis Ababa, Ethiopia); Yakob Wondarad (Ministry of Health, Addis Ababa, Ethiopia); Mohammed Ahmed (Ministry of Health, Addis Ababa, Ethiopia); Akililu Simanesew (Addis Ababa Regional Health Bureau, Addis Ababa, Ethiopia); Wondimu Ayele (Addis Ababa University, Addis Ababa, Ethiopia); Debritu Kebede (John Snow International, Addis Ababa, Ethiopia); Gobeze Neagash (John Snow International, Addis Ababa, Ethiopia); Thomas Solomon (John Snow International, Addis Ababa, Ethiopia); Afrah Mohammedsani (John Snow International, Addis Ababa, Ethiopia); J. Koku Awoonor-Williams (Ghana Health Service, Accra, Ghana); S. Patrick Kachur (Mailman School of Public Health, Columbia University, New York, USA); Ayaga A. Bawah (Regional Institute for Population Studies, University of Ghana, Accra, Ghana); Patrick Asuming (University of Ghana, Accra, Ghana); Adriana Biney (University of Ghana, Accra, Ghana); Dominic K. Atweam (Ghana Health Service, Accra, Ghana); Mallory Sheff (Mailman School of Public Health, Columbia University, New York, NY, USA); Elizabeth Jackson (Mailman School of Public Health, Columbia University, New York, NY, USA); James Phillips (Mailman School of Public Health, Columbia University, New York, NY, USA); Quinhas Fernandes (Department of Global Health, University of Washington, Seattle, WA, USA; National Directorate of Public Health, Ministry of Health, Maputo, Mozambique); Kenneth Sherr (Department of Global Health, University of Washington, Seattle, WA, USA); Isaías Ramiro (Health Alliance International, Maputo, Mozambique); Artur Gremu (Health Alliance International, Beira, Mozambique); Nelia Manaca (Health Alliance International, Beira, Mozambique); Orvalho Augusto (Department of Global Health, University of Washington, Seattle, WA, USA; Eduardo Mondlane University, Maputo, Mozambique); and Stélio Tembe (Department of Global Health, University of Washington, Seattle, WA, USA; Ministry of Health, Maputo, Mozambique).

## Supplementary Material

21-00666-Baynes-Supplement.pdf

## Translation

**Em Português**

**Barreiras e Facilitadores ao Uso de Dados para a Tomada de Decisões: A Experiência das Parcerias da African Health Initiative na Etiópia, Gana e Moçambique**

**Principais conclusões**

A criação de tempo e espaço para encontros regulares de capacitação ao nível de equipes por meio de mentoria, análise conjunta de dados e partilha de resultados, reforça o engajamento sustentável do uso de dados para a tomada de decisões ao nível local.

A política externa e os incentivos associados, levaram a um grande entusiasmo e a desafios operacionais ao nível da unidade sanitária.

O envolvimento da liderança e os recursos disponíveis ao nível subnacional até a unidade sanitária, impulsionaram a prontidão da implementação em todos os projetos. O desempenho ficou retardado nos locais onde a liderança sentiu-se despreparada para apoiar as iniciativas nacionais para expandir a gestão da saúde e os sistemas de informação. Onde a formação e a orientação subsequente foram mais abrangentes em todos os níveis do sistema de saúde, o impacto na prestação de serviços foi maior.

**Principais implicações**

Para implementar efetivamente e de forma sustentada intervenções de melhoria de dados para a tomada de decisões, os gestores das unidades de saúde devem criar ou introduzir nas reuniões de equipes já existentes, o tempo para capacitação por meio de partilha de dados conjuntos, análise e programação de melhoria.

Os gestores nacionais e subnacionais devem priorizar o engajamento e a capacidade em todos os níveis dos sistemas de saúde por meio de oportunidades de treino e orientação. De igual forma, as políticas para a melhoria das habilidades gerais da força de trabalho devem ser consideradas nos níveis individual e operacional.

**Resumo**

**Antecedente:** Três projetos de parceria da African Health Initiative (AHI) na Etiópia, Gana e Moçambique implementaram estratégias de melhoria da qualidade e avaliação de dados colhidos rotineiramente no nível dos cuidados de saúde primária e incentivar o seu uso na tomada de decisões com base em evidências. Comparamos como esses programas projetaram e executaram os dados para as estratégias de tomada de decisão (DDM); elaboramos barreiras e facilitadores para o sucesso da implementação e oferecemos recomendações para a programação futura de DDM.

**Métodos:** Pesquisadores de cada projeto escreveram colaborativamente um protocolo entre países com base nesses objetivos. Ao adaptar o Arcabouço Consolidado para a Pesquisa de Implementação (CFIR) por meio de um processo qualitativo de redução temática, eles harmonizaram as linhas de investigação sobre o desenho das respectivas estratégias de DDM e as barreiras e facilitadores de uma implementação efetiva. Conduzimos entrevistas em profundidade e discussões de grupos focais com as partes interessadas dos sistemas de cuidados de saúde primária em cada um dos países e realizamos análises temáticas em vários estágios usando o método dedutivo.

**Resultados:** A implementação efetiva do DDM dependia de os implementadores sentiram que o DDM era adaptável ao contexto, viável para testar e fácil de introduzir e manter. A política predominante e o ambiente político mais amplo no sistema de saúde, o clima de aprendizagem e capacidade de absorção para mudanças baseadas em evidências em ambientes de DDM, o envolvimento de agentes de mudança externos e líderes de mudanças internas e a promoção de oportunidades e meios para reflexão e avaliações em equipe do que funciona, influenciou o sucesso ou o fracasso das estratégias de DDM.

**Conclusão:** Oportunidades de capacitação em equipe e orientação individual levaram a uma programação eficaz das estratégias DDM. As políticas externas e os incentivos associados reforçaram isso, mas eventualmente levaram a consequências não intencionais. Engajamento da liderança e disponibilidade de recursos para agir nas recomendações; resposta às necessidades de capacitação; e facilitar a colaboração entre pares, dentro de hierarquias e em todo o sistema de saúde local provou ser crucial para o DDM, assim como encorajador à adaptação e oportunidades para o aprendizado iterativo no trabalho.

**Peer Reviewed**
