# Supplementary material for: Barriers and Facilitators to Data Use for Decision Making: The Experience of the African Health Initiative Partnerships in Ethiopia, Ghana, and Mozambique
Source: Glob Health Sci Pract. 2022 Sep 15;10(Suppl 1):e2100666. doi: 10.9745/GHSP-D-21-00666 (PMC9476487; doi:10.9745/GHSP-D-21-00666)
Supplement: 21-00666-Baynes-Supplement.pdf [file 21-00666-Baynes-Supplement.pdf]

Supplement to: AHI Partnership Collaborative for Data Use for Decision Making. Barriers and facilitators to data-use for decision-making: the experience of the African Health Initiative partnerships in Ethiopia, Ghana, and Mozambique. *Glob Health Sci Pract*. 2022;10(Suppl 1):e2100666. https://doi.org/10.9745/GHSP-D-21-00666

Supplement Table. Evidence-based interventions, by DDCF Country Project

Country	EBI	Core Components	Objectives	Intended Outcomes
Ethiopia	Connected Woreda Strategy (CWS) (linked to Ethiopia's Information Revolution Roadmap)– a tool that strengthens the performance of the health information systems at primary health care entities.[1,2]	*Accreditation process related to HIS resources, data quality and use for woreda/districts<br>*Training<br>*Mentorship	To support the delivery of quality and equitable health services through improved access to and use of quality health information for decision making at all levels.<br>Specific Objectives<br>1. To improve the quality and transformation of health information at all levels<br>2. To improve the culture of using health information for decisions at all levels<br>3. To strengthen HIS infrastructure through improved connectivity and digitalization of HIS tools<br>4. To strengthen information revolution implementation and expansion to all regions	1. M&E Systems & Capacity<br>* Approved structure, HR & other basic facilities for M&E<br>* Sufficient budget<br>*Supportive supervision ongoing<br>2. Data Quality<br>*On-time complete reporting<br>* high quality data (assessed via Lot Quality Assurance Surveys)<br>* Proper use of registers and forms, both form filling and movement of forms as needed<br>3. Administrative Data Use<br>*Functioning PMT<br>*Data analysis & dissemination is conducted<br>4. Clinic Data Use (to inform)<br>*Standards of Care<br>*Continuity of Care
Ghana	Community-based Health Planning and Services (CHPS+)[3, 4] - is a national strategy to deliver essential community-based health services in underserved sub-districts. It engages communities in planning and service	*Shorter- and longer-term training of health staff across focus areas<br>*Peer learning exchanges via the systems learning district<br>* Provision of catalytic funds to support innovations in service delivery<br>*E-Tracker	Attain the goal of reaching every community with a basic package of essential health services towards attaining Universal Health Coverage and bridging the access inequity gap	Ensure appropriate, high quality health services closer to all Ghanaians, especially those in rural or other marginalized settings.

Supplement to: AHI Partnership Collaborative for Data Use for Decision Making. Barriers and facilitators to data-use for decision-making: the experience of the African Health Initiative partnerships in Ethiopia, Ghana, and Mozambique. *Glob Health Sci Pract*. 2022;10(Suppl 1):e2100666. https://doi.org/10.9745/GHSP-D-21-00666

Country	EBI	Core Components	Objectives	Intended Outcomes
	delivery. Focus areas included community engagement, volunteer motivation, procurement of equipment and supplies, sub-district strengthening, data utilization, & capacity building.			
Mozambique	Mozambique MOH MNCH Guidelines - Comprehensive guidelines for health system managers and providers to meet the health needs and priorities of women, newborns and children. These guidelines are typically updated annually and are not published on the internet.	Guidelines provide core operating and planning instructions for health managers and providers to provide family planning and reproductive health services, maternal, newborn and child health services, promote health communications, ensure delivery of health commodities and supplies and foster health systems strengthening	Ensure the highest quality and most equitable care for all Mozambican women and their families.	Healthy Mozambican women, infants and children

[1]DOI: 10.21203/rs.3.rs-151176/v1

[2]https://static1.squarespace.com/static/57be2f5e893fc0b6f3592200/t/581d149837c581a0038c650f/1478300826355/Connected+Woreda+Program+draft++Implementation+Strategy_+20160902_NA.pdf

[3] https://www.moh.gov.gh/wp-content/uploads/2016/02/CHPS-policy-final-working-draft-for-validation.pdf

[4] https://journals.plos.org/plosone/article/file?id=10.1371/journal.pone.0226808&type=printable
